# The Effects of Dynamic Complexity on Drivers’ Secondary Task Scanning Behavior under a Car-Following Scenario

**DOI:** 10.3390/ijerph19031881

**Published:** 2022-02-08

**Authors:** Linhong Wang, Hongtao Li, Mengzhu Guo, Yixin Chen

**Affiliations:** Transportation College, Jilin University, Changchun 130022, China; wanghonglin0520@126.com (L.W.); hongtaoli1995@126.com (H.L.); lwlwyqn@163.com (Y.C.)

**Keywords:** automotive engineering, traffic safety, car-following scenario, dynamic complexity, secondary task carrying capacity, attention distribution

## Abstract

The user interface of vehicle interaction systems has become increasingly complex in recent years, which makes these devices important factors that contribute to accidents. Therefore, it is necessary to study the impact of dynamic complexity on the carrying capacity of secondary tasks under different traffic scenarios. First, we selected vehicle speed and vehicle spacing as influencing factors in carrying out secondary tasks. Then, the average single scanning time, total scanning time, and scanning times were selected as evaluation criteria, based on the theories of cognitive psychology. Lastly, we used a driving simulator to conduct an experiment under a car-following scenario and collect data on scanning behavior by an eye tracker, to evaluate the performance of the secondary task. The results show that the relationship between the total scanning time, scanning times, and the vehicle speed can be expressed by an exponential model, the relationship between the above two indicators and the vehicle spacing can be expressed by a logarithmic model, and the relationship with the total number of icons can be expressed by a linear model. Combined with the above relationships and the evaluation criteria for driving secondary tasks, the maximum number of icons at different vehicle speeds and vehicle spacings can be calculated to reduce the likelihood of accidents caused by attention overload.

## 1. Introduction

### 1.1. Background

Automotive electronic technology has developed rapidly in recent years, and drivers are becoming increasingly eager to keep in touch with others while driving and know all types of information, such as checking navigation routes, switching music, and answering phones [1]. Therefore, automobile companies are equipped with more and more electronic equipment in cars, to meet the demands of various consumers. In addition, with the improvement of the network and electronic degree of vehicles, driver’s demands for multitasking operation of the entertainment system, real-time onboard information system, and smartphones in the car are significantly increased [2]. The user interface of automotive interaction systems has become more complex, which makes these intelligent devices important causes of drivers’ distraction and, therefore, important factors that contribute to accidents [3]. With the development of sensor technology, communication technology, and the continuous proposal of the concepts of intelligence and networking, data openness and information sharing between vehicles will become an inevitable trend [4], providing a new possibility for the development of adaptive vehicle human–computer interaction systems.

The main driving behaviors include car following, lane changing, overtaking, turning, etc. Among the above driving behaviors, car following is the most basic microdriving behavior, which describes the interaction between two adjacent vehicles in the driving team on the one-way road that restricts overtaking. Car following scenario is mainly used to test the car following the performance of vehicles. In this scenario, there is basically no horizontal conflict between vehicles, and the driver only needs to take the car following behavior [5]. When drivers are changing lanes, overtaking, turning, they should pay full attention to the main driving tasks. At this time, they should not operate secondary tasks. Only in the car following scenario, the driver can scan and take over secondary tasks such as the central control screen. Therefore, we chose the car following scenario to study the effects of traffic dynamic complexity on the driver’s secondary task scanning behavior.

### 1.2. Literature Review

#### 1.2.1. Adaptive Vehicle Human–Computer Interaction Systems

The literature of adaptive human–computer interaction systems can be divided into the following two aspects. The first approach is the adaptive human–computer interaction system considering multisource perception and cognitive modeling. The system collects various nonverbal information of the driver, such as pupil size, gaze position, facial expression, driving posture, arm movement, and press strength, carries out self-regulation of the interface, and realizes in-depth communication between the interactive interface and the driver [6,7,8,9,10,11,12]. The second approach is the adaptive human–computer interaction system based on spatial three-dimensional interaction. This system uses a cooperative intelligent transportation system to collect outside information from various sensors and uses the onboard augmented reality (AR) of the windshield display screen to present the dynamic traffic information, realized through a visual display of 360° 3D virtual space around the vehicle [13,14,15,16,17,18]. As regards the design of adaptive human–computer interaction systems, researchers believe that drivers’ cognitive state and the form in which information is presented will influence the interaction effect. The adaptive adjustment of the interaction system will significantly improve the level of traffic safety. However, there is little in-depth research on the effect of drivers’ secondary task operation performances under different traffic environments and complexity levels of secondary tasks. There is a lack of quantitative research on the safety threshold of secondary task complexity under different traffic scenarios.

#### 1.2.2. Secondary Task Carrying Capacity

The secondary task carrying capacity refers to the maximum secondary task complexity that a driver can withstand during driving while avoiding excessive driving load, thus effectively reducing the probability of traffic accidents. We can analyze the impact of traffic environment and secondary tasks on drivers’ secondary task carrying capacity, taking advantage of the intelligent connected vehicle’s strong perception of the surrounding environment and adjusting the complexity of secondary tasks based on the external traffic conditions. 

The literature on the overload of drivers caused by secondary tasks mainly focuses on the impact of driving distraction on driving performance and traffic safety levels. The evaluation indicators of driving load in the subtask can be divided into driving performance indicators, response indicators, eye movement indicators, physiological and psychological indicators, etc. The driving performance is the vehicle dynamics index for the driver to judge the vehicle operation stability and safety during driving, including vehicle speed, acceleration, steering wheel angle, brake pedal position, throttle opening, etc. [19]. The response index is the response time of the driver performing a driving intention or secondary task [20]. The Eye movement index is the driver’s scanning behavior, pupil size, gaze distribution during driving, etc. [21]. Physiological and psychological indicators are used to evaluate the driver’s driving load by collecting medical indicators such as electroencephalogram (EEG), electrocardiogram (ECG), electromyogram (EMG), galvanic skin response (GSR), body temperature, respiration, and blood pressure [22].

### 1.3. Study Aim

The aim of this study was to analyze the impact of dynamic traffic environment complexity on a driver’s secondary task carrying capacity. For this purpose, first, the main influencing factors of the secondary task carrying capacity were selected by analyzing the driver’s operating process of secondary tasks under a car-following scenario. Then, a secondary task evaluation standard was established based on the relevant theories of cognitive psychology, to quantify the secondary task carrying capacity. Lastly, an experiment was designed to analyze the effect of the secondary task complexity and traffic environment on the secondary task operation performance. The study provides a scientific basis for the design and development of adaptive vehicle human–computer interaction systems in a networked car-following environment.

## 2. Materials and Methods

### 2.1. Selection of Secondary Task and Design Principles 

The central control screen is an important medium for drivers to communicate with vehicles [23]. Drivers use the central control screen to collect the vehicle status information. Meanwhile, most of the drivers’ operations during driving are completed by the central control screen [24]. A reasonable central control interactive interface can provide drivers with accurate information and convenient operation, bring drivers a comfortable driving experience, and improve driving safety. Therefore, major mainstream automobile companies have also invested considerable resources in the design of central control interactive interfaces [25]. However, drivers need to search and click on icons in the central control screen during driving, a cumbersome operation that will distract the driver’s attention, as the eyes cannot visually grasp road information and icons at the same time, which increases the likelihood of accidents [26]. As the central control screen plays an important role in driving safety, we considered searching and clicking specified icons in the central control interface as the driving secondary task. The number of icons in the secondary task *N* was selected to represent the complexity of the secondary task, and the maximum number of icons was selected to represent the secondary task carrying capacity.

**Figure 1:**
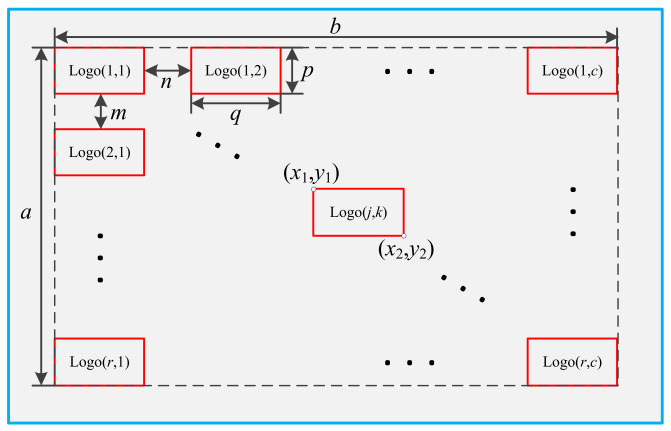
Layout of icons in interactive interface.

The icons in the central control interactive interface are generally arranged in a matrix, as shown in Figure 1. We assume that the pixel width and pixel length of the icon matrix are *a* and *b*, respectively. The number of rows and columns of the icon matrix are *r* and *c*, respectively. The pixel width and pixel length of the icon are *p* and *q*, respectively. The row spacing is *m* and the column spacing is *n*. The secondary task carrying capacity *P*(*N*) can be expressed by Equation (1) as follows:

(1) $$P\left(N\right)\;=\max\left(N\right)\;=\max\left(rc\right)$$

In this paper, the design of the secondary task icon matrix need to satisfy the following three principles:

(1) The number of rows and columns of the icon matrix should be consistent with the size of the background.

The central control screen generally adopts a rectangular design, and mainstream manufacturers adopt horizontal screen placement. However, the central control screens of some manufacturers can be adjusted to vertical placement. In order to make full use of the space in the interactive interface, rows *r* and columns *c* of the icon matrix in the interface need to satisfy a correlation that can be expressed by Equation (2) as follows:

(2) $$\left\{\begin{array}{l} r\leq c\leq r+2\;\;a<b\\ c\leq r\leq c+2\;\;a\geq b \end{array}\right.$$

(2) There should be an intelligent match between the icon area and the number of icons in the interface.

To make full use of the space in the interactive interface, it is necessary to scale the length and width of a single icon when increasing or decreasing the number of icons in the interface. Two cases of *p* = *q* and *p* ≠ *q*, respectively, need to be addressed.

If *p* = *q*, the icon matrix may overflow from the right or below side of the background. Therefore, it is necessary to adjust the preset row and column spacing. The input parameters are *r*, *c*, *m*, and *n* of the icon matrix, while the output parameters are *p* or *q*, *m*_a_, and *n*_a_. The calculation method can be expressed as follows:

(3) $$p=\left\{\begin{array}{l} \;\frac{b-n\left(c-1\right)}{c}\;\;a\cdot c>b\cdot r\\ \frac{a-m\left(r-1\right)}{r}\;\;a\cdot c\leq b\cdot r \end{array}\right.$$

(4) $$m_{a}=\left\{\begin{array}{l} \;\frac{a-p\;\cdot \;r}{r+1}\;\;a\cdot c>b\cdot r\\ m\;\;\;\;a\cdot c\leq b\cdot r \end{array}\right.$$

(5) $$n_{a}=\left\{\begin{array}{l} \;n\;\;\;\;a\cdot c>b\cdot r\\ \frac{b-p\;\cdot \;c}{c+1}\;\;\;a\cdot c\leq b\cdot r \end{array}\right.$$

If *p* ≠ *q*, the overflow of the icon matrix from the interface background can be avoided by calculating the appropriate *p* and *q*; therefore, it is unnecessary to adjust the preset row and column spacing. The input parameters are *r*, *c*, *m*, and *n* of the icon matrix, and the output parameters are *p* and *q*. The calculation method can be expressed as follows:

(6) $$p=\frac{a-\left(r-1\right)m}{r}$$

(7) $$q=\frac{b-\left(c-1\right)n}{c}$$

(3) The icons are arranged symmetrically in the center of the interface.

To ensure that the icons in the interface shall be arranged in the form of center alignment, the position of the icon logo(*j*,*k*) in the *j*-th row and the *k*-th column of the icon matrix can be expressed by its upper left corner coordinates (*x*_1_(*j*,*k*), *y*_1_(*j*,*k*)) and lower right corner coordinates (*x*_2_(*j*,*k*), *y*_2_(*j*,*k*)). The calculation method can be expressed as follows:

(8) $$x_{1}\left(j,k\right)\;=0.5\left(a-r\cdot p-\left(r-1\right)\cdot m\right)+\left(j-1\right)\cdot \left(p+m\right)$$

(9) $$x_{2}\left(j,k\right)\;=0.5\left(a-r\cdot p-\left(r-1\right)\cdot m\right)+j\cdot p+\left(j-1\right)\cdot m$$

(10) $$y_{1}\left(j,k\right)\;=0.5\left(b-c\cdot q-\left(c-1\right)\cdot n\right)+\left(k-1\right)\cdot \left(q+n\right)$$

(11) $$y_{2}\left(j,k\right)\;=0.5\left(b-c\cdot q-\left(c-1\right)\cdot n\right)+k\cdot q+\left(k-1\right)\cdot n$$

### 2.2. Evaluation Model of Secondary Task Carrying Capacity under a Car-Following Scenario

**Figure 2:**
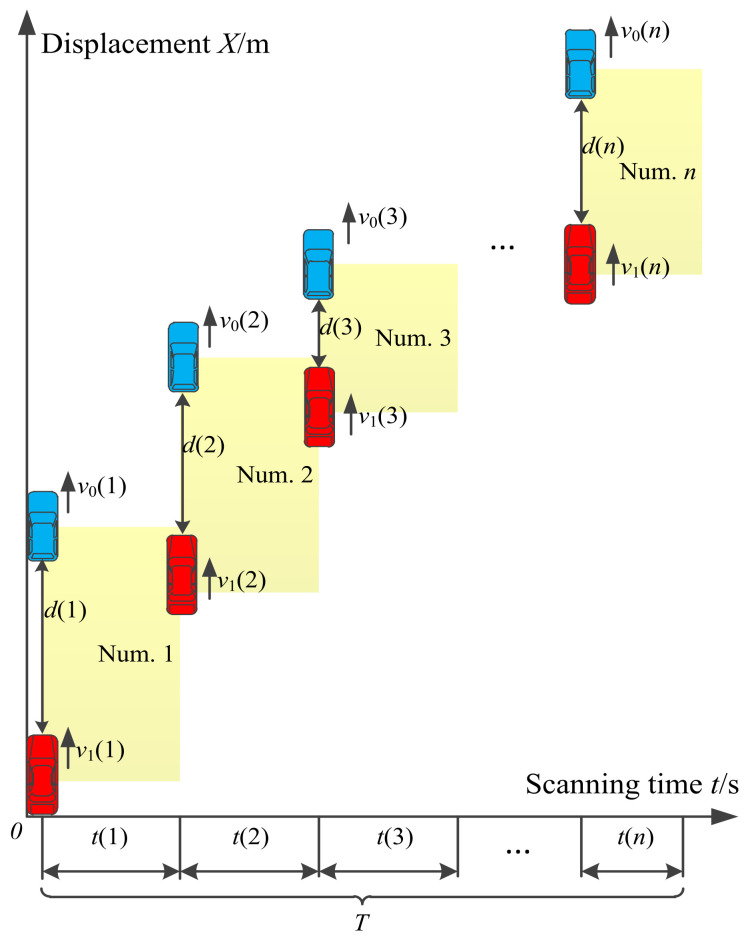
The travel process of the driver’s vehicle and the front vehicle in car-following scenario when the driver is operating the secondary task.

The traveling process of the driver’s vehicle and the front vehicle in a car-following scenario when the driver is operating the secondary task is shown in Figure 2, in which the driver’s vehicle is red, and the front vehicle is blue. The secondary task appears at 0 s. In the car-following scenario, the process of drivers operating the secondary task can be described as follows: Drivers collect information on the traffic environment and vehicle status through visual, auditory, and tactile sensory channels. When the secondary task occurs, they make decisions on the secondary task combined with driving experience. If drivers judge that the current traffic environment is not complicated, they will distribute attention from the main driving task to the secondary task. If the complexity of the traffic environment exceeds drivers’ carrying capacity, they will stop operating the secondary task and take measures to reduce the complexity of the traffic environment until they can continue operating the secondary task.

In the above process, the driver’s sight will be away from the road ahead when he scans the secondary task, which is the most likely cause of traffic accidents. We assumed that the driver needs to scan *n* times until completing the secondary task, where the driver’s *i*-th scan is referred to as the “num.*i*”. We assumed that when the driver starts the *i*-th scanning, the initial distance between the driver’s vehicle and the front vehicle is *d*(*i*), the speed of the front vehicle is *v*_0_(*i*), the speed of the driver’s vehicle is *v*_1_(*i*), the single scanning time of the secondary task is *t*(*i*), the complexity of the secondary task is *C*, and the total scanning time required for the driver to operate the secondary task is *T*. The total scanning time is affected by the vehicle speed and spacing between the two vehicles and the complexity of the secondary task; therefore, *T* can be expressed by Equation (12) as follows:

(12) $$T=f\left(v_{0}\left(i\right),v_{1}\left(i\right),d\left(i\right),C\right)$$

When the driver completes the secondary task, *t*(*i*) and *T* need to satisfy the relationship shown in Equation (13).

(13) $$\sum_{i=1}^{n}t\left(i\right)=T$$

According to NHTSA-2010-0053 issued by the National Highway Traffic Safety Administration and the relevant theories of cognitive psychology [27,28,29], the evaluation criteria for driving secondary tasks include the following three aspects:

(1) The average single scanning time (including sight transfer time) should not exceed 2.2 s;
(2) The scanning times of a single secondary task should not exceed four times;
(3) The total scanning time of a single secondary task should not exceed 15 s.

Therefore, the constraint of drivers’ secondary task carrying capacity *P*(*N*) can be expressed by Equation (14) as follows:

(14) $$\left\{\begin{array}{l} s.t.\;\;\mathrm{mean}\left(t\right)\leq 2.2\\ s.t.\;\;n\leq 4\\ s.t.\;\;T\leq 15 \end{array}\right.$$

## 3. Experimental Design and Data Acquisition

### 3.1. Experimental Equipment

A UC-win/road driving simulator was used in the indoor environment to provide safe and controlled research scenarios. The hardware of the driving simulator included a high-performance computer, three LCD screens working together to display the video information of the driving scene, a Logitech G29 steering wheel with an accelerator, and a brake pedal kit. A tablet computer with a touch function was used to display secondary tasks. A Tobii Pro Glasser 2 eye tracker was used to collect drivers’ eye movement data during the experiment. The driver was considered to start scanning the secondary task when their gaze area changed from the front road to the central control interface. When the gaze area changed from the central control interface to the front road, it was considered that the subject had ended the scanning process of the secondary task.

**Figure 3:**
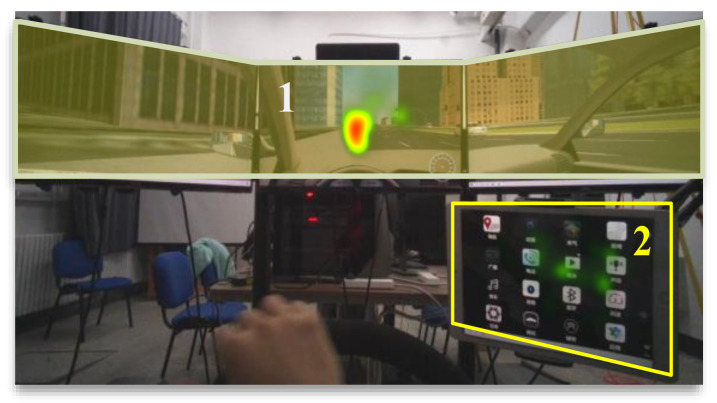
Heat map of participants’ eye movements in the two areas.

To collect data on the average single scanning time, total scanning time, and scanning times, the driver’s gaze area was divided into two parts. Area 1 was the road ahead, while area 2 was the secondary task interface. The heat map of participants’ eye movements in the two areas is shown in Figure 3; in this figure, red indicates a greater likelihood of viewing in that location.

The time from the gaze point leaving area 1 to returning to area 1 again in each group of the experiment was regarded as the time for a single scanning [30]. Tobii Pro Lab software was used to extract the single scanning time, scanning times, and total scanning time. The calculation method of single scanning time is as follows: The time when the gaze point leaving area 1 is recorded as the start time of single scanning *t_b_*(*i*), and the time when the gaze point returning to area 1 is recorded as the end time of single scanning *t_e_*(*i*). The calculation formula of single scanning time *t*(*i*) can be expressed by Equation (15) as follows:

(15) $$t\left(i\right)\;=t_{e}\left(i\right)-t_{b}\left(i\right)$$

### 3.2. Experimental Scheme

**Figure 4:**
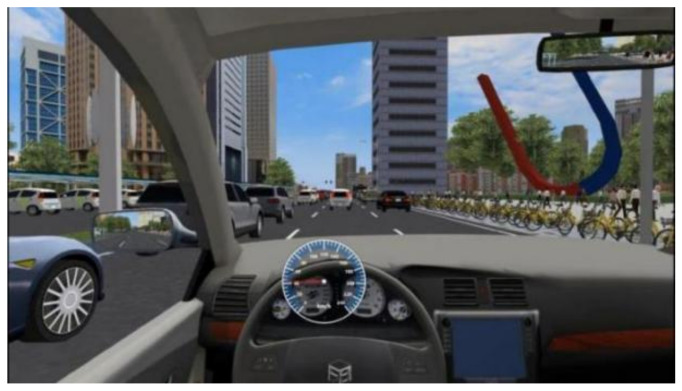
Car-following driving scenario.

To collect data on the scanning behavior of drivers under different traffic environments and secondary tasks in a car-following scenario, a simulated driving experiment was conducted under a stable car-following scenario (i.e., acceleration difference between the front vehicle and the driver’s vehicle should be maintained between −0.6 m·s^−2^ and 0.6 m·s^−2^ [31]). The road type in the scenario was a two-way, six-lane urban road (as shown in Figure 4), the length of the road was 20 km, the driver’s vehicle traveled in the middle lane, the traffic flow on both sides of the middle lane was 300 veh·h^−1^, and the average speed of the traffic flow was 60 km·h^−1^.

**Figure 5:**
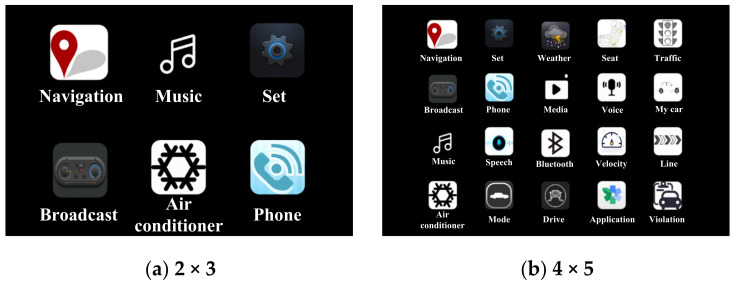
Part icon layouts used in the experiment.

The tablet computer was placed horizontally. The icons in the experiment were designed with the icons in the real vehicle interaction interface as the template, where, *a* is 650, *b* is 900. The icon was square. *r* was taken as 2, 3, and 4, respectively. *c* was taken as 2, 3, 4, 5, and 6, respectively. *m* and *n* were all taken as 20 pix. According to Equation (2), a total of 9 groups of icons layouts with different numbers were obtained by the combination of *r* and *c*. The layout parameters of different numbers of icon matrices were calculated according to Equations (3)–(5), which are shown in Table 1. Part icon layouts were used in the experiment, as shown in Figure 5.

According to the speed limit of urban roads, the front vehicle speeds were taken as 20 km·h^−1^, 30 km·h^−1^, 40 km·h^−1^, 50 km·h^−1^, 60 km·h^−1^, and 70 km·h^−1^, respectively. It traveled in the middle lane at a constant speed. At each vehicle speed, the spacing distances between the driver’s driving vehicle and the front vehicle were taken as 10 m, 15 m, 20 m, 25 m, 30 m, and 35 m, respectively. Drivers followed the front vehicle, and collisions and lane changes were not allowed in the whole process.

### 3.3. Data Collection

A total of 30 drivers—18 men and 12 women—participated in the study. The drivers were aged between 22 and 35 (mean = 26.17, standard deviation = 2.72). To be able to maintain a relatively stable operation ability to complete the experiment, all drivers needed to conduct a 20-min simulated driving operation exercise before the experiment, which included being familiar with the vehicle braking performance, driving environment, and speed control ability.

During the experiment, the driver drove the vehicle while continuously following the front vehicle and completed secondary tasks of nine icon layouts at six different vehicle speeds and six different vehicle spacings, respectively. Therefore, each driver had to complete 324 (6 × 6 × 9) groups of tests. When the investigator randomly pressed the trigger switch of the secondary task, the system randomly selected an icon in the touch interface as the secondary task and played the prompt voice “please open ××!”. After the prompt voice ended, the driver would find and click the secondary task. After the secondary task was completed, the investigator recorded the driver’s operation time of the secondary task. The driver’s experiment finished when they completed all tests.

## 4. Results

**Figure 6:**
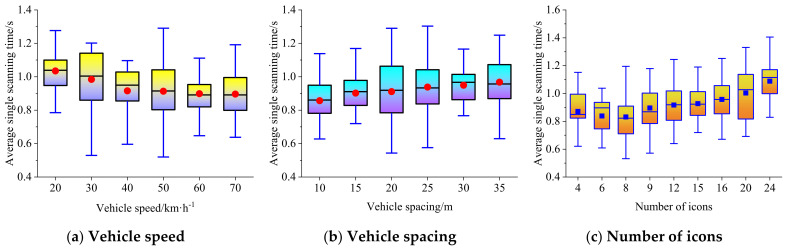
Boxplot of average single scanning time at different vehicle speeds, vehicle spacings, and the number of icons.

**Figure 7:**
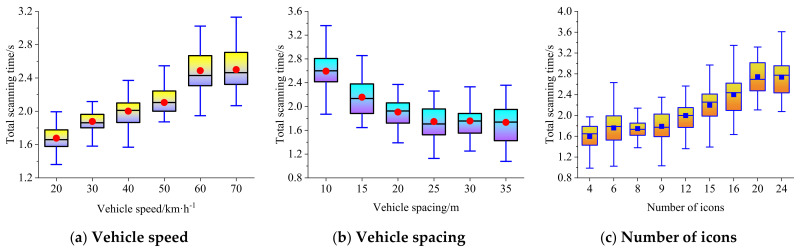
Boxplot of total scanning time at different vehicle speeds, vehicle spacings, and the number of icons.

**Figure 8:**
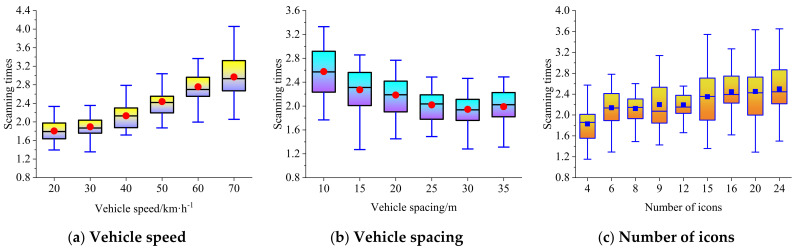
Boxplot of scanning times at different vehicle speeds, vehicle spacings, and the number of icons.

The dynamic complexity is related to the vehicle speed and spacing. The boxplots of average single scanning time, total scanning time, and scanning times at different vehicle speeds, vehicle spacings, and the number of icons are shown in Figure 6, Figure 7 and Figure 8, respectively.

We used SPSS data analysis software (IBM, Armonk, NY, USA) to analyze the effects of vehicle speed and spacing on the secondary task carrying capacity. 

### 4.1. Average Single Scanning Time

Figure 6 shows that when the traffic environment becomes more and more complex (e.g., with an increase in vehicle speed or a decrease in vehicle spacing), the average single scanning time of the driver for the secondary task will show a downward trend, which indicates that with higher levels of dynamic complexity, the driver increases the proportion of attention attributed to the main task of driving to ensure safety. When the complexity of the secondary task becomes larger, and the external traffic environment remains unchanged (the number of icons increases), the average single scanning time of drivers shows an upward trend. The relationship between the average single scanning time and the vehicle speed can be expressed by a negative logarithmic regression model (R^2^ = 0.962), the relationship between the average single scanning time and the vehicle spacing can be expressed by a positive logarithmic regression model (R^2^ = 0.992), and the relationship between the average single scanning time and the number of icons can be expressed by a positive linear regression model (R^2^ = 0.735). The relationship between vehicle speed, vehicle spacing, the number of icons, and the average single scanning time meeting the upper limit of 95% confidence interval can be expressed by the multivariate nonlinear fitting model shown in Equation (16).

(16) $$\mathrm{mean}\left(t\right)\;=0.079\ln\left(d\right)-0.108\ln\left(v\right)+0.01N+1.749$$

### 4.2. Total Scanning Time

Figure 7 shows that, when the traffic environment becomes more and more complex, the total scanning time of the driver for the secondary task shows an upward trend, and when the traffic environment remains unchanged, the complexity of the secondary task becomes higher, and the driver’s total scanning time also shows an upward trend. Meanwhile, the relationship between the total scanning time and vehicle speed can be expressed by an exponential regression model (R^2^ = 0.985), the relationship between the total scanning time and vehicle spacing can be expressed by a negative logarithmic regression model (R^2^ = 0.903), and the relationship between the total scanning time and the number of icons can be expressed by a positive linear regression model (R^2^ = 0.922). The relationship between vehicle speed, vehicle spacing, the number of icons, and the total scanning time meeting the upper limit of 95% confidence interval can be expressed by the multivariate nonlinear fitting model shown in Equation (17).

(17) $$T=-0.421\ln\left(d\right)-1.258\exp\left(0.0083v\right)+0.08N+5.111$$

### 4.3. Scanning Times

Figure 8 shows that, with the increase in vehicle speed or the decrease in vehicle spacing, the scanning times of the driver for the secondary task show an upward trend. When the number of icons increases, but the external traffic environment remains unchanged, the driver’s scanning times also show an upward trend. In this trend, the relationship between scanning times and vehicle speed can be expressed by an exponential regression model (R^2^ = 0.979), the relationship between scanning times and vehicle spacing can be expressed by a negative logarithmic regression model (R^2^ = 0.974), and the relationship between scanning times and the number of icons can be expressed by a positive linear regression model (R^2^ = 0.783). The relationship between vehicle speed, vehicle spacing, the number of icons, and the scanning times meeting the upper limit of 95% confidence interval can be expressed by the multivariate nonlinear fitting model shown in Equation (18).

(18) $$n=\frac{T}{\mathrm{mean}\left(t\right)}=\frac{-0.421\ln\left(d\right)-1.258\exp\left(0.0083v\right)+0.08N+5.111}{0.079\ln\left(d\right)-0.108\ln\left(v\right)+0.01N+1.749}$$

## 5. Discussion

It is necessary to deeply analyze the driver’s attention contention process under the joint influence of subtask and main driving task. This research accurately determined the scientific basis for subtask settings under different levels of dynamic complexity in traffic environments. The average single scanning time for secondary tasks shows a downward trend when the vehicle speed increases or the vehicle spacing decreases. In addition, when the number of icons in the secondary task increases, the average single scanning time shows an upward trend. This indicates that when the complexity of a traffic environment becomes higher, the driver actively increases the proportion of attention assigned to the main driving task, to ensure traffic safety. However, a high level of complexity of the secondary task will weaken this effect, resulting in the secondary task carrying capacity of drivers exceeding the safety threshold, easily leading to traffic accidents.

Substituting Equations (16)–(18) into Equation (14), the relationship between the number of icons, vehicle speed, and vehicle spacing can be expressed by Equation (19) as follows:

(19) $$\left\{\begin{array}{l} \;0.079\ln\left(d\right)-0.108\ln\left(v\right)+0.01N+1.749\leq 2.2\\ \;\frac{-0.421\ln\left(d\right)-1.258\exp\left(0.0083v\right)+0.08N+5.111}{0.079\ln\left(d\right)-0.108\ln\left(v\right)+0.01N+1.749}\leq 4\\ -0.421\ln\left(d\right)-1.258\exp\left(0.0083v\right)+0.08N+5.111\leq 15 \end{array}\right.$$

Excessive icons increase the risk of traffic accidents during driving. To ensure driving safety, the number of icons should not exceed 24. Combined with Equation (19), the driver’s secondary task carrying capacity of icons can be expressed by Equation (20) as follows:

(20) $$\left\{\begin{array}{l} \;P\left(N\right)\leq \left(-0.079\ln\left(d\right)+0.108\ln\left(v\right)+0.451\right)/0.01\\ \;P\left(N\right)\leq \left(0.737\ln\left(d\right)+1.258\exp\left(0.0083v\right)-0.432\ln\left(v\right)+1.885\right)/0.04\\ 0\leq P\left(N\right)\leq 24 \end{array}\right.$$

The sensitivity analysis of the impact of vehicle speed and vehicle spacing on the secondary task carrying capacity shows that with the decrease in vehicle speed or increase in vehicle spacing, the impact of the two influencing factors on the secondary task carrying capacity decreases gradually, and there is a marginal decreasing effect. Compared with vehicle speed, the impact of vehicle spacing on the secondary task carrying capacity is more sensitive. Therefore, increasing the vehicle spacing has a more significant effect on improving the secondary task carrying capacity of drivers. 

**Figure 9:**
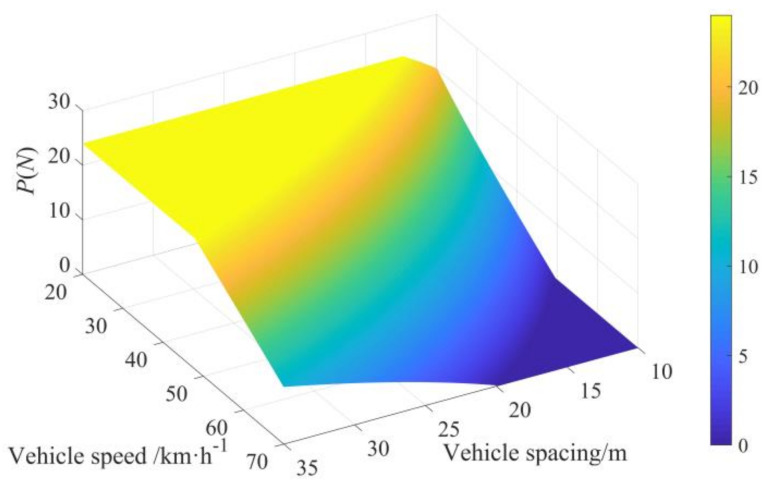
Secondary task carrying capacity of icons *P*(*N*) at different vehicle speeds and vehicle spacings.

**Figure 10:**
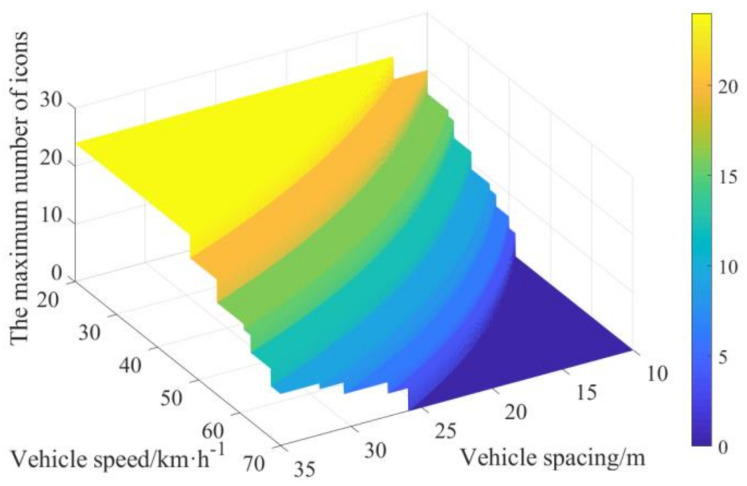
The maximum number of icons at different vehicle speeds and vehicle spacings.

The secondary task carrying capacity with the vehicle speed in the range of 20–70 km·h^−^^1^ and the vehicle spacing in the range of 10–35 m is calculated by Equation (20) and shown in Figure 9. The secondary task carrying capacity is rounded by the constraints of Equation (2). The maximum number of icons at different vehicle speeds within the range of 20–70 km·h^−^^1^ and vehicle spacings within the range of 10–35 m is calculated as shown in Figure 10, and the specific values are shown in Table 2.

As evident from Figure 10 and Table 2, to ensure that the complexity of the secondary task does not exceed the driver’s carrying capacity on the premise that the central control interface can display icons, the vehicle spacing should not be less than 10 m, 13.5 m, 18.6 m, and 25.7 m when the vehicle speed is 40 km·h^−^^1^, 50 km·h^−^^1^, 60 km·h^−^^1^, and 70 km·h^−^^1^, respectively. 

## 6. Conclusions

To reduce traffic accidents caused by driving distraction, we studied the impact of dynamic complexity on secondary tasks carrying capacity under different traffic scenarios. We selected vehicle speed and vehicle spacing as the influencing factors in carrying out secondary tasks. The average single scanning time, total scanning time, and scanning times were selected as the evaluation criteria, considering the theories of cognitive psychology. A simulated driving experiment was conducted as an example, to evaluate the performance of secondary tasks under different levels of dynamic complexity. The following conclusions were drawn from this study:

(1) The relationship between vehicle speed, vehicle spacing, the number of icons, and average single scanning time can be expressed by a negative logarithmic model, a positive logarithmic model, and a positive linear model, respectively. The relationship between vehicle speed, vehicle spacing, the number of icons, and total scanning time can be expressed by a positive exponential model, a negative logarithmic model, and a positive linear model, respectively. The relationship between vehicle speed, vehicle spacing, the number of icons, and scanning times can be expressed by a positive exponential model, a negative logarithmic model, and a positive linear model, respectively. Combined with the above relationships and the evaluation criteria for driving secondary tasks, we calculated the maximum number of icons at different vehicle speeds and vehicle spacings. In this way, we can dynamically adjust the number of icons in the central control screen under the car-following scenario, to avoid the occurrence of traffic accidents caused by attention overload.
(2) The average single scanning time for secondary tasks shows a downward trend when the vehicle speed increases or the vehicle spacing decreases. In addition, when the number of icons in the secondary task increases, the average single scanning time shows an upward trend. This reveals that when the complexity of the traffic environment becomes higher, the driver actively increases the proportion of attention allocated to the main driving task, to ensure traffic safety. However, a highly complex secondary task will weaken this effect, resulting in the secondary task carrying capacity of drivers exceeding the safety threshold, thus easily leading to traffic accidents.
(3) With the decrease in vehicle speed or the increase in vehicle spacing, the impact of these two influencing factors on the secondary task carrying capacity decreases gradually, leading to a marginal decreasing effect. Compared with vehicle speed, the impact of vehicle spacing on the secondary task carrying capacity is more sensitive. To ensure that the complexity of the secondary task does not exceed the driver’s carrying capacity on the premise that the central control interface can display icons, the vehicle spacing should not be less than 10 m, 13.5 m, 18.6 m, and 25.7 m when the vehicle speed is 40 km·h^−^^1^, 50 km·h^−^^1^, 60 km·h^−^^1^, and 70 km·h^−^^1^, respectively.

In a future study, a questionnaire will be conducted on drivers to obtain the use frequency of applications in the central control screen during driving; then, the importance of different applications will be sorted according to the use frequency. When some information must be removed from displays, the system should gradually remove the applications with lower importance and keep the applications often used by drivers as much as possible. In this way, the missing symbols will not cause problems, and user experience will not be affected while ensuring safe driving. 

## Figures and Tables

**Table 1:** The layout parameters of different number of icon matrices.

Icons Matrix Dimension	*p*/pix	*m*_a_/pix	*n*_a_/pix	*N*
2 × 2	315	20	90	4
2 × 3	287	26	20	6
2 × 4	210	77	20	8
3 × 3	204	20	72	9
3 × 4	204	20	17	12
3 × 5	164	40	20	15
4 × 4	148	20	62	16
4 × 5	148	20	27	20
4 × 6	134	23	20	24

**Table 2:** The maximum number of icons *N_m_* at different vehicle speeds *v* and vehicle spacings *d*.

		v/km·h^−1^	20	30	40	50	60	70
	N_m_							
d/m								
**10**			20	12	4	0	0	0
**15**			24	16	12	6	0	0
**20**			24	24	16	12	6	0
**25**			24	24	20	16	9	0
**30**			24	24	24	16	12	6
**35**			24	24	24	20	16	9

## Data Availability

The data presented in this study are available in the paper.

## References

[1] Iio K., Guo X., Lord D. (2021). Examining driver distraction in the context of driving speed: An observational study using disruptive technology and naturalistic data. Accid. Anal. Prev..

[2] Ma J., Gong Z., Tan J., Zhang Q., Zuo Y. (2020). Assessing the driving distraction effect of vehicle HMI displays using data mining techniques. Transp. Res. Part F Traffic Psychol. Behav..

[3] Ahangari S., Jeihani M., Ardeshiri A., Rahman M.M., Dehzangi A. (2021). Enhancing the performance of a model to predict driving distraction with the random forest classifier. Transp. Res. Rec..

[4] Ning Z., Zhang K., Wang X., Guo L., Hu X., Huang J., Hu B., Ricky Y.K.K. (2020). Intelligent edge computing in internet of vehicles: A joint computation offloading and caching solution. IEEE Trans. Intell. Transp. Syst..

[5] Ding N., Lu Z., Jiao N., Lu L. (2021). Quantifying effects of reverse linear perspective as a visual cue on vehicle and platoon crash risk variations in car-following using path analysis. Accid. Anal. Prev..

[6] Biondi F., Alvarez I., Jeong K.A. (2019). Human-vehicle cooperation in automated driving: A multidisciplinary review and appraisal. Int. J. Hum.-Comput. Interact..

[7] Duric Z., Gray W.D., Heishman R., Li F., Rosenfeld A., Schoelles M.J., Schunn C., Wechsler H. (2002). Integrating perceptual and cognitive modeling for adaptive and intelligent human-computer interaction. Proc. IEEE..

[8] Ekman F., Johansson M., Sochor J. (2017). Creating appropriate trust in automated vehicle systems: A framework for HMI design. IEEE Trans. Hum.-Mach. Syst..

[9] Ulahannan A., Jennings P., Oliveira L., Birrell S. (2020). Designing an adaptive interface: Using eye tracking to classify how information usage changes over time in partially automated vehicles. IEEE Access..

[10] Pinotti D., Piccinini G.F.B., Tango F. (2014). Adaptive human machine interface based on the detection of driver’s cognitive state using machine learning approach. Intell. Artif..

[11] Oviedo T.O., Haque M.M., King M., Demmel S. (2018). Driving behaviour while self-regulating mobile phone interactions: A human-machine system approach. Accid. Anal. Prev..

[12] de Naurois C.J., Bourdin C., Bougard C., Vercher J. (2018). Adapting artificial neural networks to a specific driver enhances detection and prediction of drowsiness. Accid. Anal. Prev..

[13] Wang X., Zheng X., Chen W., Wang F. (2020). Visual Human-Computer Interactions for Intelligent Vehicles and Intelligent Transportation Systems: The State of the Art and Future Directions. IEEE Trans. Syst. Man Cybern. Syst..

[14] Blomeyer D., Schulte-Gehrmann A.L. (2019). Surface innovations for interiors of future vehicles. ATZ Worldw..

[15] Klumpp M., Zijm H. (2019). Logistics innovation and social sustainability: How to prevent an artificial divide in Human-Computer Interaction. J. Bus. Logist..

[16] Klumpp M., Hesenius M., Meyer O., Ruiner C., Gruhn V. (2019). Production logistics and human-computer interaction—state-of-the-art, challenges and requirements for the future. Int. J. Adv. Manuf. Technol..

[17] Riegler A., Wintersberger P., Riener A., Holzmann C. (2019). Augmented Reality Windshield Displays and Their Potential to Enhance User Experience in Automated Driving. i-com J. Interact. Media.

[18] Rahmati Y., Talebpour A., Mittal A., Fishelson J. (2020). Game Theory-Based Framework for Modeling Human–Vehicle Interactions on the Road. Transp. Res. Rec..

[19] Niu J., Wang X., Liu X., Wang D., Qin H., Zhang Y. (2019). Effects of mobile phone use on driving performance in a multiresource workload scenario. Traffic Inj. Prev..

[20] Lin R., Liu N., Ma L., Zhang T., Zhang W. (2019). Exploring the self-regulation of secondary task engagement in the context of partially automated driving: A pilot study. Transp. Res. Part F Traffic Psychol. Behav..

[21] Faure V., Lobjois R., Benguigui N. (2016). The effects of driving environment complexity and dual tasking on drivers’ mental workload and eye blink behavior. Transp. Res. Part F Traffic Psychol. Behav..

[22] Hensch A., Rauh N., Schmidt C., Hergeth S., Naujoks F., Krems J.F., Keinath A. (2020). Effects of secondary tasks and display position on glance behavior during partially automated driving. Transp. Res. Part F Traffic Psychol. Behav..

[23] Noble A.M., Miles M., Perez M.A., Guo F., Klauer S.G. (2021). Evaluating driver eye glance behavior and secondary task engagement while using driving automation systems. Accid. Anal. Prev..

[24] Metz B., Landau A., Just M. (2014). Frequency of secondary tasks in driving–Results from naturalistic driving data. Saf. Sci..

[25] Metz B., Schömig N., Krüger H.P. (2011). Attention during visual secondary tasks in driving: Adaptation to the demands of the driving task. Transp. Res. Part F Traffic Psychol. Behav..

[26] Guo B., Jin L., Sun D., Shi J., Wang F. (2019). Establishment of the characteristic evaluation index system of secondary task driving and analyzing its importance. Transp. Res. Part F Traffic Psychol. Behav..

[27] Khawaja M.A., Chen F., Marcus N. (2012). Analysis of collaborative communication for linguistic cues of cognitive load. Hum. Factors.

[28] Chen F., Ruiz N., Choi E., Epps J., Khawaja M.A., Taib R., Yin B., Wang Y. (2013). Multimodal behavior and interaction as indicators of cognitive load. ACM Trans. Interact. Intell. Syst..

[29] Khawaja M.A., Chen F., Marcus N. (2014). Measuring cognitive load using linguistic features: Implications for usability evaluation and adaptive interaction design. Int. J. Hum.-Comput. Interact..

[30] Hu H., Cheng M., Gao F., Sheng Y., Zheng R. (2020). Driver’s Preview Modeling Based on Visual Characteristics through Actual Vehicle Tests. Sensors.

[31] Rakha H. (2009). Validation of Van Aerde’s simplified steadystate car-following and traffic stream model. Transp. Lett..

